# Assessment of Coupling between Trans-Abdominally Acquired Fetal ECG and Uterine Activity by Bivariate Phase-Rectified Signal Averaging Analysis

**DOI:** 10.1371/journal.pone.0094557

**Published:** 2014-04-23

**Authors:** Daniela Casati, Tamara Stampalija, Konstantinos Rizas, Enrico Ferrazzi, Cristina Mastroianni, Eleonora Rosti, Mariachiara Quadrifoglio, Axel Bauer

**Affiliations:** 1 Department of the Woman, Mother and Neonate, Children's Hospital Vittore Buzzi, Biomedical and Clinical Sciences School of Medicine University of Milan, Milan, Italy; 2 Unit of Prenatal Diagnosis, Institute for Maternal and Child Health, IRCSS Burlo Garofolo, Trieste, Italy; 3 Department of Cardiology, Munich University Clinic, DZHK (German Centre for Cardiovascular Research), Ludwig-Maximilians University, Munich, Germany; Baylor College of Medicine, United States of America

## Abstract

Couplings between uterine contractions (UC) and fetal heart rate (fHR) provide important information on fetal condition during labor. At present, couplings between UC and fHR are assessed by visual analysis and interpretation of cardiotocography. The application of computerized approaches is restricted due to the non-stationarity of the signal, missing data and noise, typical for fHR. Herein, we propose a novel approach to assess couplings between UC and fHR, based on a signal-processing algorithm termed bivariate phase-rectified signal averaging (BPRSA).

**Methods:**

Electrohysterogram (EHG) and fetal electrocardiogram (fECG) were recorded non-invasively by a trans-abdominal device in 73 women at term with uneventful singleton pregnancy during the first stage of labor. Coupling between UC and fHR was analyzed by BPRSA and by conventional cross power spectral density analysis (CPSD). For both methods, degree of coupling was assessed by the maximum coefficient of coherence (C_PRSA_ and C_RAW_, respectively) in the UC frequency domain. Coherence values greater than 0.50 were consider significant. C_PRSA_ and C_RAW_ were compared by Wilcoxon test.

**Results:**

At visual inspection BPRSA analysis identified coupled periodicities in 86.3% (63/73) of the cases. 11/73 (15%) cases were excluded from further analysis because no 30 minutes of fECG recording without signal loss was available for spectral analysis. Significant coupling was found in 90.3% (56/62) of the cases analyzed by BPRSA, and in 24.2% (15/62) of the cases analyzed by CPSD, respectively. The difference between median value of C_PRSA_ and C_RAW_ was highly significant (0.79 [IQR 0.69–0.90] and 0.29 [IQR 0.17–0.47], respectively; p<0.0001).

**Conclusion:**

BPRSA is a novel computer-based approach that can be reliably applied to trans-abdominally acquired EHG-fECG. It allows the assessment of correlations between UC and fHR patterns in the majority of labors, overcoming the limitations of non-stationarity and artifacts. Compared to standard techniques of cross-correlations, such as CPSD, BPRSA is significantly superior.

## Introduction

Labor represents a stress test for the fetus. Indeed, fetal hypoxia and asphyxia during labor constitute a possible cause of cerebral palsy and other neurological complications in childhood and adulthood[1]. Therefore, surveillance of the fetal wellbeing by intra-partum monitoring is of crucial importance. Analysis of fetal heart rate (fHR) recordings provides valuable information about the fetal homeostasis. In fact, fHR variability is an indirect mirror of an oxygenation and integrity of the autonomous nervous system[2], and can be altered by specific intrinsic or extrinsic stimuli. The uterine contractions (UC) generate transient reductions in utero-placental blood flow to the fetus, which represent a strong external stimulus to fetal homeostasis capable to modify fHR[3]. The inter-relationship between UC and fHR might provide important information about the functional status of fetal autonomous nervous system during labor. In the vast majority of deliveries, the intermittent hypoxia to which the fetus is exposed during UC is well tolerated, but in some cases this is not the case. Thus, the evaluation of the fetal response to UC as an early marker of fetal distress and the recognition of fHR patterns that could reveal compromised fetal oxygenation are of major clinical interest[4].

Currently, the most common approach to assess the interaction between fHR and UC is based on the interpretation of standard cardiotocographic (CTG) method[4], although it has several disadvantages: 1) only qualitative visual interpretation is feasible, without quantitative evaluation; 2) there is a high intra- and inter-observer variability[5], [6]; and 3) the interpretation depends on the expertise of the physician. For these reasons, an objective computerized approach to assess the couplings between UC and fHR would be of great clinical value.

Herein, we propose a novel analysis method of coupling between UC and fHR based on a signal-processing algorithm, first applied in adult cardiology, termed bivariate phase-rectified signal averaging (BPRSA)[7]–[9] and applied to trans-abdominally acquired uterine electromiography and fetal ECG (fECG). This method overcomes the limitation of non-stationary signal and background noise typical for fHR signal. The aim of the study was to: 1) evaluate the coupling between UC and fHR by BPRSA analysis; and 2) compare BPRSA analysis to other analytic approaches.

## Materials and Methods

This is a prospective study in which women with a singleton uneventful pregnancy at term were simultaneously monitored during labor with standard CTG and a trans-abdominal electrocardiogram/electrohysterogram (ta-fECG/EHG) recording device (Monica AN24, Monica Healthcare, Nottingham, UK). The results of ta-fECG/EHG recordings were blinded to the managing personnel, and all clinical decisions were taken based on standard CTG. Women were recruited at Buzzi Children's Hospital, Biomedical and Clinical Science School of Medicine, University of Milan, according to a research protocol named ‘*Trans-abdominal fetal ECG and mioelectro-hysterogram (EHG) in term and post-term pregnancy and delivery*’ approved by the ICP (Istituti Clinici di Perfezionamento) Ethical Committee. All participants gave their informed written consent.

Fetal ECG and uterine EHG were recorded trans-abdominally at 300 Hz for the whole length of labor and stored for off-line analysis. The Uterine Activity was extracted from the slow-wave of the electro-hysterogram (EHG), i.e. its envelope. The envelope was obtained by low-pass filtering the rectified fast-wave of the EHG. The R-R intervals were measured from QRS complexes. The envelope of the EHG and the R-R intervals were averaged every two seconds to extract the profile of the UC and the fHR.

To eliminate ectopic beats and compensatory/non-compensatory pauses, HR beats values greater than 200 beats per minutes (bpm) and less than 50 bpm were filtered out.

**Figure 1:**
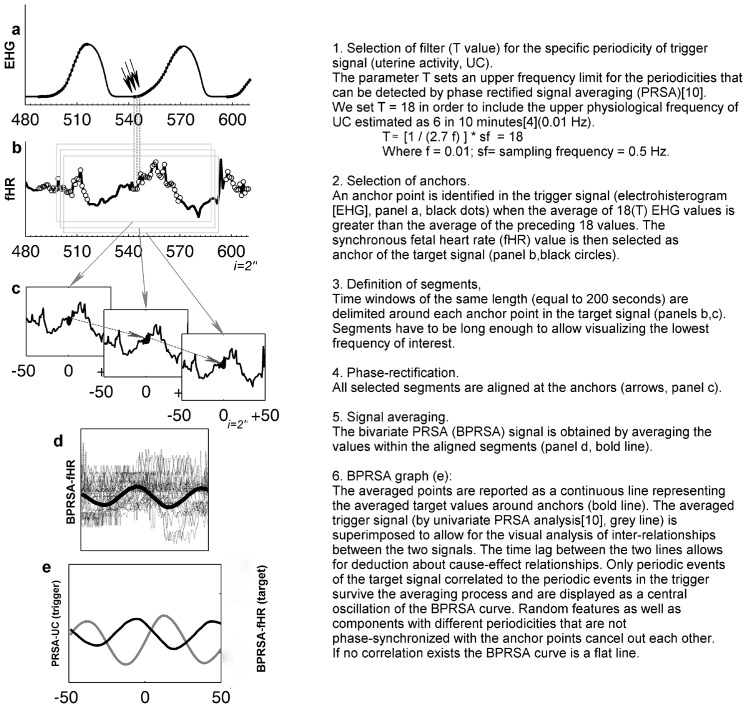
Description of the Bivariate Phase Rectified Signal Averaging (BPRSA) method. Panel **a**) Uterine Activity by EHG (trigger signal): each data point is the average of raw signal every 2 seconds. Each black dot represents an anchor point (EHG increases). Panel **b**) fetal heart rate by ECG (target signal). The empty circles represent anchor points derived from the EHG signal. Panel **c**) Segments of fetal heart rate to average, centered around the anchor point (time windows of 200 seconds, each data point(i) is the average of fHR every 2 seconds). Panel **d**) phase rectification at the anchor points and signal averaging. Panel **e**) BPRSA graph representing PRSA transformation of Uterine Activity (grey line) and BPRSA transformation of fetal heart rate (bold line).

For the first step of the analysis, 30 minutes long EHG-fECG time-series were used for the BPRSA analysis from recordings of the active stage of labor (from 3 cm of dilation to complete dilation, before pushing). For this step, the only criteria for selection was the presence of 30 minutes with <50% of signal loss. The 30 minutes length was chosen in order to be long enough to contain an adequate number of contractions (>3 per ten minutes), but not too long in order to avoid high signal loss, major non-stationarities, noise and significant changes in fetal autonomic reactivity. In each tracing the inter-relations between UC (as *trigger signal*), as detected by increasing values of EHG (EHG^↑^), and synchronous values of fHR (as *target signal*) were analyzed by means of the BPRSA algorithm. An experienced clinician in BPRSA analysis (DC) classified the graphic outputs of BPRSA. Figure 1 summarizes the steps of BPRSA analysis for these two obstetrical variables. For further readings refer to Bauer[8] and Schumann[7].

The second step was the quantification of coupling between the *trigger* and *target* signals by means of the magnitude squared coherence method applied to cross power spectral density (CPSD) analysis. The frequency composition of the signals was calculated using a Welch-spectral analysis. First we computed spectral analysis on the EHG signal to obtain the UC frequency domain of each case. Then, we calculated the maximum coefficient of coherence (C_PRSA_) at the specific UC frequency domain considering significant coherence values greater than 0.50[11], [12]. The coefficient of coherence obtained in this way was compared to visual analysis.

In the third step, only 30-minutes time-series free of ECG signal loss were selected (where available) for each fECG-EHG recording. This was performed in order to apply the strict criteria of spectral analysis and minimize the effects of artifacts and background noise. After eliminating ectopic beats as previously described, a linear interpolation was applied to obtain continuous signal tracings and linear trends were removed. CPSD analysis was then performed on the original EHG-fECG signals and on their PRSA transformations to allow a comparison. The maximum coefficients of coherence (C_RAW_ and C_PRSA_, respectively), at the specific UC frequency domain, were calculated for each case. In order to estimate the ability of BPRSA analysis to identify couplings between UC and fHR, the differences between coherence values were tested by means of non-parametric tests (paired Wilcoxon sign rank test). Two-tailed p-values less than 0.05 were considered statistically significant. All analyses were performed using Matlab (R2011b, MathWorks) and SPSS 19 (IBM Corp, Armonk, NY). Fig.1.

## Results

### Detection and quantification of couplings between UC and fHR by BPRSA

**Figure 2:**
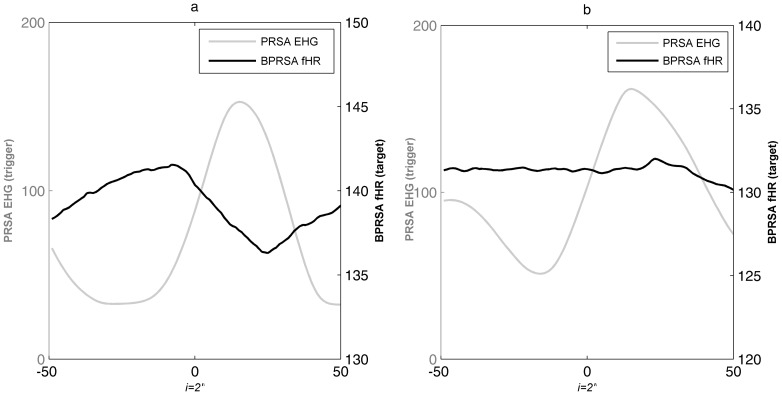
Example of bivariate phase rectified signal averaging (BPRSA) graphs: grey line: univariate PRSA transformation of electrohysterogram increases (EHG^↑^) time series; black line: bivariate PRSA transformation of fetal heart rate (fHR) values in correspondence to EHG^↑^. The time axis, centered around time 0 (aligned anchors), allows to observe the oscillation of the signals before and after the triggering event (time windows of 200 seconds, each data point(i) is the average of PRSA-signals every 2 seconds). Panel a: presence of coupling (black line oscillation); panel b: absence of coupling (flat black line).

The BPRSA analysis was applied on 73 ta-fECG-EHG tracings recorded during the first stage of labor. At a visual interpretation of the BPRSA graphs, a central oscillation of BPRSA signal, indicating the presence of coupling between UC and fHR, was visible in 86.3% (63/73) cases (Figure 2a). On contrary, in 13.7% (10/73) cases no deflection of BPRSA signal was visually identified suggesting the absence of correlation between UC and fHR signals (Figure 2b).

**Figure 3:**
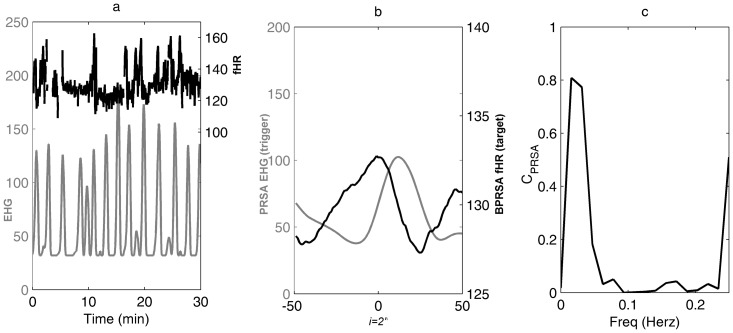
Detection of cross-correlations by cross power spectral density (CPSD) analysis applied to PRSA transformed signals: a ) 30-minutes electrohysterogram and fetal heart rate (EHG-fHR) recording; **b**) BPRSA graph denoting presence of coupled periodicities between uterine PRSA (grey line) and fHR BPRSA (black line); **c**) CPSD analysis of PRSA transformed signals. The coefficient of coherence C_PRSA_ shows a significant correlation at the UC frequency domain (C_PRSA_ = 0.81).

The frequency domain of UC obtained by spectral analysis showed in each tracing a peak around 0.0069 Hz (median, interquartile range [IQR] 0.0061–0.0078 Hz). The median value of the maximum coefficients of coherence (C_PRSA_) between the univariate PRSA transformation of EHG increases (EHG^↑^) and BPRSA transformation of fHR time series calculated by CPSD at the individual UC frequency domain, was 0.76 (IQR 0.60–0.85). A coherence coefficient >0.50 (denoting presence of significant cross-correlation between the two signals frequency spectra) was found in 85% (62/73) cases (Figure 3). When comparing the results of coupling assessed by visual interpretation and CPSD analysis there was a disagreement in overall 6/73 cases: in 5/63 cases the presence of coupling assessed by visual interpretation was not confirmed by CPSD analysis (median C_PRSA_ 0.38, IQR 0.34–0.47); and in 1/10 case of absence of coupling assessed by visual interpretation the coherence coefficient turned out significant at CPSD analysis (C_PRSA_ = 0.64).

### Comparison of BPRSA with conventional cross spectral analysis

**Figure 4:**
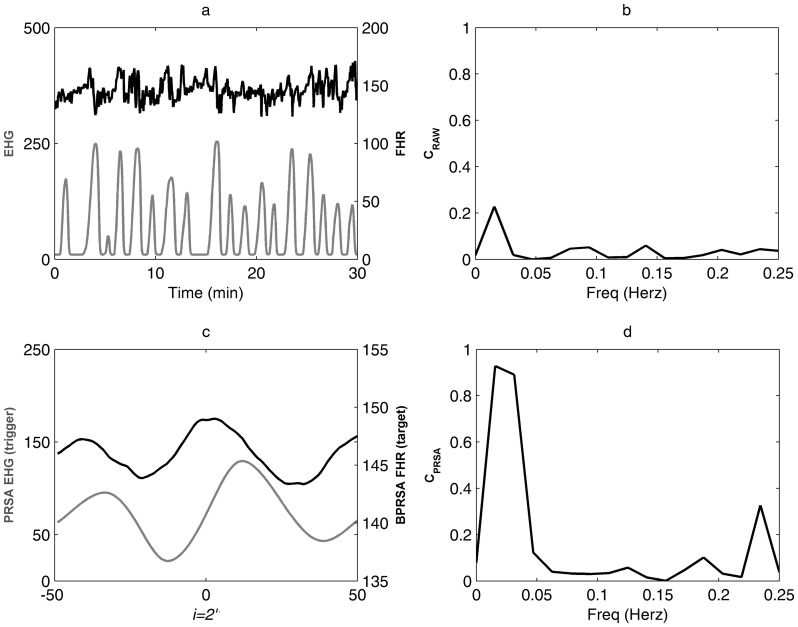
Comparison of BPRSA with conventional cross spectral analysis: example of detection of coupling by BPRSA. **a**) 30 minutes recording of electrohysterogram (EHG, grey line) and fetal heart rate (fHR, black line) free of signal loss; **b**) cross power spectral density (CPSD) analysis of original signals, C_RAW_<0.5 at the uterine contraction (UC) frequency domain; **c**) BPRSA graph showing coupled periodicities; d) CPSD analysis of PRSA signals, the C_PRSA_>0.5 at the UC frequency domain proved a significant correlation.

**Figure 5:**
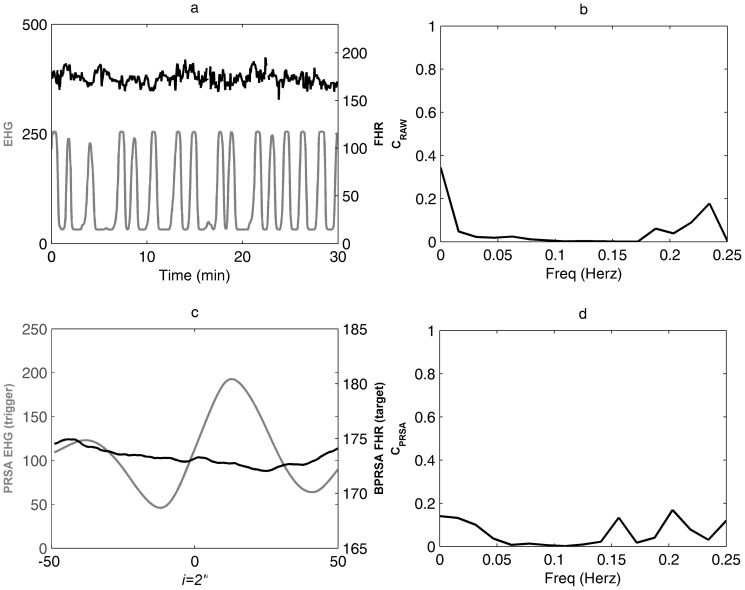
Comparison of BPRSA with conventional cross spectral analysis; example of absence of coupling by BPRSA. **a**) 30 minutes recording of electrohysterogram (EHG, grey line) and fetal heart rate (fHR, black line) free of signal loss; **b**) cross power spectral density (CPSD) analysis of original signals, C_RAW_<0.5 at the uterine contraction (UC) frequency domain; **c**) BPRSA graph displaying absence of coupled periodicities (flat BPRSA signal, black line); **d**) CPSD analysis of PRSA signals, the C_PRSA_<0.5 at the UC frequency domain proved the absence of significant correlation.

In 11 of the 73 (15%) cases a continuous recording without ECG signal loss for at least 30 minutes was not available. Thus, these cases were excluded from the spectral analysis, and the remaining 62 cases were analyzed in the third step of the study. At CPSD analysis of the original signals (EHG-fHR time series), the maximum coefficient of coherence (C_RAW_) calculated at the individual UC frequency domain was 0.29 (median, IQR 0.17–0.47) (Figures 4 and 5; panels a and *b*). The coherence coefficient was >0.50 in 24.2% (15/62) of cases. In contrast, BPRSA transformation (Figures 4 and 5, panel c) and subsequent CPSD analysis (Figures 4 and 5, panel d) lead to maximum coefficients of coherence (C_PRSA_) of 0.79 (median; IQR 0.69–0.90). The coherence coefficient by BPRSA was >0.50 in 90.3% (56/62) cases. In 6 of the 62 cases (9.7%) the coefficient of coherence was not significant. Of note, in all these 6 cases also the C_RAW_ was not significant. Similarly, all cases with significant C_RAW_ showed also a significant C_PRSA_, while in the majority of the cases (41/47) with C_RAW_ less than 0.50 C_PRSA_ resulted significant.

**Figure 6:**
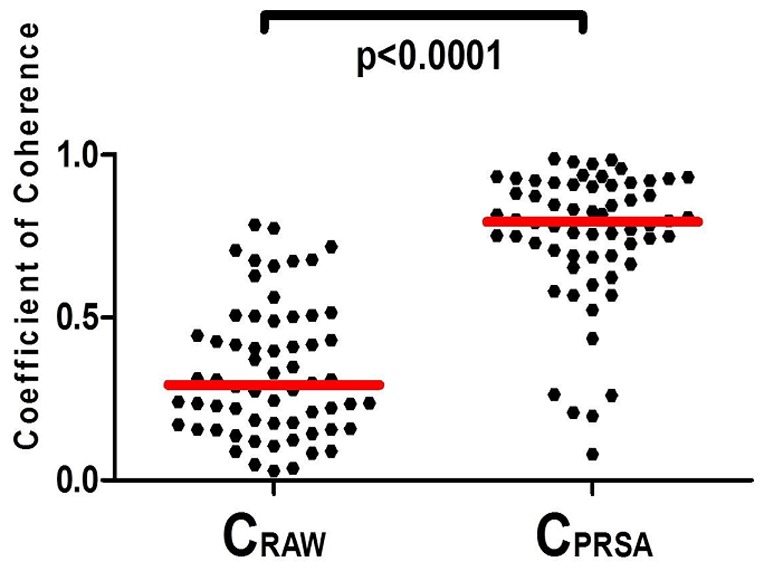
Comparison between coefficients of coherence provided by conventional cross-spectral analysis (C_RAW_, median 0.29, interquartile range 0.17–0.49) and by bivariate Phase-Rectified Signal Averaging (C_PRSA_ median 0.79, interquartile range 0.69–0.91); p<0.0001.

The difference between the coefficients of coherence obtained by CPSD analysis of original signals (C_RAW_) and CPSD analysis of PRSA-transformed signals (C_PRSA_) was highly significant (p<0.0001; Figure 6).

## Discussion

The analysis of inter-relations between two or more biological signals provides important insight into the regulation processes of a system and, thus, might be used for surveillance or diagnostic purposes. In adult cardiology, the assessment of couplings between arterial blood pressure and heart rate is an example where cross-correlations between two biological signals are of high clinical relevance[11], [12]. The identification of specific periodic patterns of HR, undetectable at visual inspection, is feasible by means of spectral analysis[13]–[18]. Indeed, fetal acidosis relates to specific frequency-band changes related to autonomous nervous system modulation of fHR[16], [17]. Although the spectral analysis of the fHR has been widely investigated[14], [16]–[18], its clinical application during labor is largely limited by the need for stationary and artifacts-free signals. Warrick et al. proposed a nonparametric system-identification model to explore uterine pressure-fHR dynamics in terms of impulse response function that proved successful in detecting fetal distress[19], [20]. However, each methodology, that uses the Doppler as the origin of fHR signal, might be intrinsically limited due to stochastic error and feto-maternal HR confusion inherent to the methodology[21]. Moreover, external pressure transducer for UC assessment is known to underestimate the duration of contractions, and to vary accordingly to the contact of the pressure sensor and/or abdominal wall thickness[19]. Thus, the development of a method capable to assess the association between UC and fHR patterns in objective and reproducible way, and simultaneously applicable on non-stationary and noisy signal, would be of great clinical interest. In the last years, the PRSA technique[10] has emerged as an alternative approach capable to overcome these shortcomings. This analytical method is a powerful tool for the detection and quantification of quasi-periodic oscillations of HR, both in the cardiologic[22], [23] and obstetric fields[24].

We proved that BPRSA, an extension of the original method, can be applied to trans-abdominally acquired recordings of fECG and EHG, and is robust to artifacts and missing data[7], [9]. Therefore, BPRSA analysis represents a genuine new approach to investigate modifications of fHR periodicities coinciding with uterine activity. Following the BPRSA transformation of raw electrophysiological signals, a clear-cut visual correlation between fHR patterns and UC periodicities was observed in the majority of the cases. This proves that in most laboring women there is a close interdependence between UC and fHR variations that reflects the homeostasis of the system. Nevertheless, as learnt from the CTG experience, any qualitative assessment has intrinsic limitations. Thus, we aimed to verify the reliability of the method through a mathematical quantitative computation by a conventional method of coherence evaluation as the magnitude squared coherence method applied to CPSD analysis of the PRSA transformed signals. This mathematical computation confirmed the presence of coupling in the majority of the cases in which the operator detected a significant BPRSA oscillation. Nevertheless, in 8.2% of cases there was a disagreement between visual assessment and CPSD analysis. These findings highlight the key importance of a computer-base support to the visual interpretation of the interrelations between two complex signals.

The comparison of PRSA transformed signals to conventional methods of cross-correlations evaluation (such as CPSD analysis of original signals) showed significantly better performance of PRSA method. The latter detected coupling between fHR and UC in patients where traditional CPSD analysis failed to identify any correlation. This finding confirms that the analysis of non-stationary and noise-polluted signals by CPSD method alone, without extensive pre-processing of raw data, is limited by major shortcomings, and suggests that the application of PRSA method prior to spectral analysis significantly improves the quality of the spectra[7], [9]. In addition, the lack of a continuous signal might represent an insuperable limitation for traditional spectral analysis (i.e. in our cohort 15% of tracings) that was rode over by the application of BPRSA method.

### Strenghts and limitations of the study

This is a descriptive methodological study, first of its kind in the obstetric field, which wants to lay the groundwork for future clinical application. The evaluation of biological and clinical significance of different patterns of coupling (or its absence) between UC and fHR assessed by BPRSA method goes beyond the purposes of this methodological research and remains to be determined. These evaluations are the object of ongoing research.

## Conclusions

We described a novel computer-based approach, BPRSA, that can be reliably applied to trans-abdominally acquired EHG-fECG, for the assessment of the couplings between UC and fHR patterns. We proved that in the majority of labors fHR is strongly influenced by uterine activity. The BPRSA method offers the advantage to overcome the non-stationarity, missing data and noise, typical of biological signals. Compared to standard computerized assessment technique of cross-correlations, such as cross power spectral density analysis, BPRSA is significantly superior.
